# Ultrasound-based assessment of gastric volume in enterally fed critically ILL patients

**DOI:** 10.1186/2197-425X-3-S1-A609

**Published:** 2015-10-01

**Authors:** J Skornik, H Bomberg, N Weidner, A Meiser, T Volk, HV Groesdonk

**Affiliations:** Department of Anesthesiology, Critical Care and Pain Medicine, University Hospital Homburg/Saar, Homburg, Germany

## Introduction

Enteral feeding via gastric tube has become part of routine care for critically ill patients. However, studies investigating the optimal volume and duration of nutrition in these patients are lacking. Moreover, underestimation of residual gastric volume due to gastric emptying disorders provides increased pulmonary aspiration risk. In this context, gastric ultrasonography allows qualitative and quantitative assessment of gastric contents and volume in nonobese and obese patients using an ultrasound based measurement of the cross-sectional area (CSA) of the antrum.

## Objectives

In this prospective study, we evaluated the accuracy of ultrasound based gastric volume measurement in critically ill patients. Additionally, we evaluated a new ultrasound pocket device for its usefulness in measuring gastric volume.

## Methods

Gastric volume was determined before and after a passive reflux-test by means of measuring the cross-sectional area (CSA) of the antrum. During the reflux-test, the gastric tube was opened for 5 minutes and the reflux-volume was documented. The measurements were performed in all patients first by the readily available Vivid i Ultrasound machine with a curved-array transducer and second with the V-scan maschine, both from General Electric (GE Healthcare, Little Chalfont, UK).

## Results

Up to now, we performed 15 measurements with both devices and identified 6 patients with more than 100 ml of gastric volume (110-193 ml). Interestingly, in all of these 6 patients no reflux was detectable during the reflux-test. Regarding reproducibility of the gastric volume measurements using the Vivid i ultrasound machine, there were no significant differences between the measurements before and after the reflux-test (75 ± 56 ml vs. 78 ± 54 ml; p = 0.61). The same was true for the pocket device (65 ± 58 ml vs. 59 ± 50 ml; p = 0.64).

## Conclusions

This study underlines the usefulness of ultrasound based gastric volume measurement in critically ill patients with standard ultrasound and pocket devices, respectively.

